# Supplementary material for: Novel CYP11A1-Derived Vitamin D and Lumisterol Biometabolites for the Management of COVID-19
Source: Nutrients. 2022 Nov 11;14(22):4779. doi: 10.3390/nu14224779 (PMC9698837; doi:10.3390/nu14224779)
Supplement: Supplementary file 1 [file nutrients-14-04779-s001.zip › nutrients-1891231-supplementary.pdf]

Figure S1 The change in the expression of *ACE2* and *TMPRSS2* in keratinocyte HaCaT cell line after treatment with listed vitamin D and lumisterol metabolites at 2 × 10-7 M [39].

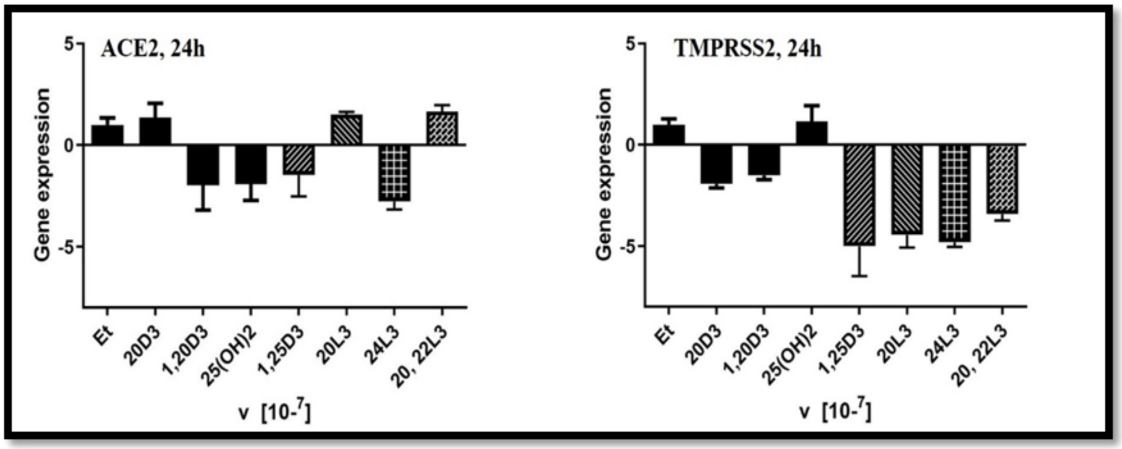

HaCaT keratinocytes were cultured in TPP tissue culture petri dishes (Ø60mm, 22.1cm²) in DMEM containing 10% cFBC (charcoal FBS) to reach semiconfluency (60-80%) and then exposed to 2 X 10^{-7} M compounds or a corresponding concentration of ethanol solvent and were used for RNA isolation (RNAeasy Micro kit, Qiagen) after 24 h of incubation. RNA isolated from HaCaT cells was submitted for cDNA synthesis (High Capacity cDNA Reverse Transcription Kit with RNase Inhibitor, Applied Biosystems) following the manufacturers' protocols. RT-PCR was carried out using Cyber green, in triplicates. *Cyclophilin B* (*CIC-B*) was used as internal control. Primer sequences are listed as:

ACE2: (L: TCCAGTACTGTAGATGGTGC; R: CTCCTTCTCAGCCTTGTTGC),

TMPRSS2: (L: CCTCTTAACAATCCATGGCATTG ; R: GGGCAGACACACTGGTTTCA),

CIC-B: (L: TGTGGTGTTTGGCAAAGTTC; R: GTTTATCCCGGCTGTCTGTC).

Data was analyzed one way ANOVA using GraphPad Prism statistical software and inhibition of *ACE2* expression was significant ($p<0.05$) for 1,20(OH)2D3, 25(OH)D3, 1,25(OH)2D3 and 24(OH)L3, while *TMPRSS2* was significantly inhibited ($p<0.05$) by 20(OH)D3, 1,20(OH)2D3, 1,25(OH)2D3, 20(OH)L3, 24(OH)L3 and 20,23(OH)2L3.
